# Activation of p47phox as a Mechanism of Bupivacaine-Induced Burst Production of Reactive Oxygen Species and Neural Toxicity

**DOI:** 10.1155/2017/8539026

**Published:** 2017-06-08

**Authors:** Yu-jie Li, Wei Zhao, Xu-jiao Yu, Feng-xian Li, Zi-ting Liu, Le Li, Shi-yuan Xu

**Affiliations:** Department of Anesthesiology, Zhujiang Hospital, Southern Medical University, Guangzhou 510282, China

## Abstract

Bupivacaine has been shown to induce neurotoxicity through inducing excessive reactive oxygen species (ROS), but the underlying mechanism remains unclear. NOX2 is one of the most important sources of ROS in the nervous system, and its activation requires the membrane translocation of subunit p47phox. However, the role of p47phox in bupivacaine-induced neurotoxicity has not been explored. In our in vitro study, cultured human SH-SY5Y neuroblastoma cells were treated with 1.5 mM bupivacaine to induce neurotoxicity. Membrane translocation of p47phox was assessed by measuring the cytosol/membrane ratio of p47phox. The effects of the NOX inhibitor VAS2870 and p47phox-siRNA on bupivacaine-induced neurotoxicity were investigated. Furthermore, the effect of VAS2870 on bupivacaine-induced neurotoxicity was assessed in vivo in rats. All these changes were reversed by pretreatment with VAS2870 or transfection with p47phox-siRNA in SH-SY5Y cells. Similarly, pretreatment with VAS2870 attenuated bupivacaine-induced neuronal toxicity in rats. It is concluded that enhancing p47phox membrane translocation is a major mechanism whereby bupivacaine induced neurotoxicity and that pretreatment with VAS2870 or local p47phox gene knockdown attenuated bupivacaine-induced neuronal cell injury.

## 1. Introduction

Local anesthetics (LAs), including bupivacaine, are commonly used for regional anesthesia and postoperative pain relief. However, application of LAs may also induce neurological injury to patients. The rate of neurological complications occurring during spinal anesthesia regardless of whether lidocaine or bupivacaine had been used was about 2.2/10,000 to 14.4/10,000 according to a survey in France [1]. It is noteworthy that the rate of permanent neurological injury ranged from 0–4.2 : 10,000 and 0–7.6 : 10,000 after spinal and epidural anesthesia, respectively [2].

LAs exhibit time- and dose-dependent toxicity to a variety of tissues and cells, including nerves and neurons [3–6]. LAs can be neurotoxic even in a normal dose or a relatively lower dose [5, 7]. Among LAs, bupivacaine is the most widely used and documented. Although full recovery of sensory motor function was observed after intrathecal administration of 0.5% and 5% bupivacaine in a rat model, histopathological abnormalities were detected after 5% bupivacaine administration [8]. However, the mechanism by which bupivacaine induced neurotoxicity remains unclear. Studies in mouse N2a cells have shown that bupivacaine induced burst production of reactive oxygen species (ROS), leakage of lactate dehydrogenase (LDH), decline mitochondrial potential, nuclear condensation, and cell apoptosis that were associated with inhibition of the AKT/PI3K pathway [9, 10]. Our previous study also showed that bupivacaine induced human SH-SY5Y cell ROS burst, DNA damage, mitochondrial dysfunction, ER stress (endoplasmic reticulum stress) [5, 11, 12]. These pathways were all involved with ROS burst. Thus, the burst production of ROS seems to be one of the key points in bupivacaine-induced cytotoxicity. However, most of the currently available studies were focused on the injury induced by overproduction of ROS, while the source or mechanism of bupivacaine-induced ROS production was largely unknown.

ROS plays an important role in cell proliferation, differentiation, migration, and host defense [13]. Excessive ROS may irreversibly destroy or alter the function of proteins, lipids, nuclear acids, membranes, and organelles, which may lead to apoptosis [13, 14]. Several enzymes in the body are capable of producing ROS, such as xanthine oxidase, cytochrome P450 oxidases, lipoxygenases, uncoupled nitric oxide synthase (NOS), NADPH oxidases (NOX), and the mitochondrial electron transport chain [13, 15]. Except for NOX, the other sources of ROS are byproducts triggered by the increase level of cellular ROS [15, 16]. In contrast, NOX produces ROS as their primary and sole function [13, 15, 16]. Therefore, NOX may be prime target candidates for neuroprotection against bupivacaine-induced neurotoxicity.

NOX is a family of proteins generating ROS when activated. NOX2, also known as gp91phox, is the main source of cytoplasmic ROS and plays an important role in the disease and injury of the nervous system [13, 17, 18]. In intact HAEC cells transfected with siNOX1, siNOX2, siNOX4, or siNOX5, only siNOX2 completely abrogated Ang-II-stimulated production of cytoplasmic O_2_^.−^ [19]. Inhibition or knockdown of NOX2 improved the outcome of the spinal cord injury model and ischemic stroke model in mice [17, 18]. NOX2 is a multiprotein complex assembled from a membrane-spanning flavocytochrome b558 (composed of gp91phox and p22phox) and four cytoplasmic components (p47phox-p67phox-p40phox and Rac1 [GDP-bound protein]) [20]. The activation of NOX2 needs the migration of cytosolic subunits to the membrane [13]. The initial and essential factor of activation is p47phox phosphorylation and membrane translocation [20, 21]. Moreover, p47phox is the unique subunit which only exists in NOX2 isoform [13, 21]. NOX2 activity has been shown to be consistent with the level of cytoplasmic O_2_^.−^ and the translocation level of p47phox [22, 23]. Given the important role of ROS in bupivacaine-induced generation, we, therefore, hypothesized that bupivacaine may induce membrane translocation of p47phox to activate NOX2, which induces overproduction of ROS and subsequently leads to cell injury.

## 2. Materials and Methods

### 2.1. Cell Culture and Drug Treatments

SH-SY5Y cells were cultured in DMEM/F12 medium (Gibco, Grand Island, NY) with 10% fetal bovine serum (BI, Israel), 100 U/ml penicillin, and 100 *μ*g/ml streptomycin (Gibco, Grand Island, NY) in a humidified 5% CO_2_ incubator at 37°C. The medium was renewed every other day.

In our previous study [12], bupivacaine treatment (0, 0.5, 1.0, 1.5, 2.0, and 2.5 mM) for 24 h decreased cell viability in a dose-dependent manner ranging from 0% to 92.73 ± 12.18%, and the IC_50_ of bupivacaine was 1.5 mM. So we chose this concentration for further study. SH-SY5Y cells were treated with bupivacaine at the concentration of 1.5 mM. Bupivacaine-treated SH-SY5Y cells were divided into 6 groups: Con (control group),1 h (treated with bupivacaine for 1 hour), 3 h (treated with bupivacaine for 3 hours),6 h (treated with bupivacaine for 3 hours and incubated with fresh basic medium for 3 hours), 12 h (treated with bupivacaine for 3 hours and incubated with fresh basic medium for 9 hours), and 24 h (treated with bupivacaine for 3 hours and incubated with fresh basic medium for 21 hours).

VAS2870 is a novel small molecular inhibitor which is reported to selectively inhibit the activity of NOX [15, 24]. SH-SY5Y cells were pretreated with VAS2870 at a concentration of 10 *μ*M [15, 24] for 30 minutes before exposure to bupivacaine. Cells were divided into 4 groups: Con (control group), Bup (treated with bupivacaine), VAS (treated with VAS2870), VAS + Bup (bupivacaine-treated cells pretreated with VAS2870).

### 2.2. Transfection of SH-SY5Y Cells with siRNA

To deplete protein levels of the catalytic subunits of NADPH oxidase, we inhibited the expression of p47phox using small interfering RNA (siRNA, applied by Ribobio, China), and a negative control (NC siRNA) was used. The siRNA sequence for p47phox was as follows: sense: 5′-ACGCGCACAGCATCCACCA-3′ and antisense: 5′-UGGUGGAUGCUGUGCGCGU-3′. SH-SY5Y cells were transfected with p47phox-siRNAs in DMEM/F12 medium containing lipo3000 transfection reagent (Invitrogen, Massachusetts, USA) as the protocol provided by Invitrogen. The transfected cells were harvested 72 hours (h) after transfection for protein assay by Western blotting and ROS measurement as well as TUNEL staining for apoptosis.

Cells were divided into 4 groups: Con (cells transfected with NC), Bup (cells transfected with NC and treated with bupivacaine), p47phoxsi (cells transfected with p47phox-siRNA), p47phoxsi + Bup (cells transfected with p47phox-siRNA and treated with bupivacaine).

### 2.3. Animal Model and Experimental Design

Animals were provided by the Animal Experiment Center of Southern Medical University. All experiments were done according to the guidelines on ethical standards approved by the Animal Experimentation Ethics Committee of Southern Medical University. Experiments were conducted on 24 male SD rats (body weight [BW], 150–200 g). Rats were randomly divided into 4 groups (*n* = 6 each group): sham operation group (Con), VAS group (intravenously injected with VAS2870), Bup group (intrathecally injected with 3% bupivacaine), VAS + Bup group (pretreatment with VAS2870 30 min prior to intrathecally injection of bupivacaine). The rats were anesthetized by inhalation of sevoflurane. The subarachnoid space was cannulated using a polyethylene tube (0.6 × 700 mm) through the midpoint of the hip joint (L6 plane) [6]. The tip of the catheter was 2 cm from caudal to the L5 level. The other end of the catheter was fixed in the subcutaneous tissue to avoid displacement. If the catheter was in the subarachnoid space, there was light yellow transparent cerebrospinal fluid flowing from the catheter. After the catheter was fixed, each rat was intrathecally injected with 30 *μ*l 2% lidocaine. If the rats occurred lower limb paralysis and pain disappearing after intrathecally injection with lidocaine, the animal model was successfully established. The rats were allowed to recover fully, and 3% bupivacaine was injected after the catheterization procedure. In addition, the NOX inhibitor VAS2870 was injected intravenously at the concentration of 2 mg/kg [25] 30 minutes prior to intrathecal injection with bupivacaine. Rats showing symptoms of traumatic nerve damage were excluded from the study.

### 2.4. Paw Withdrawal Threshold Assay

This study was performed at the same time of the day and by the same experimenter. The tests were performed before and 24 hours after the intrathecal injection with bupivacaine. Each rat was placed in a plastic cage with a wire mesh bottom which allowed full access to the paws. After cage exploration and major grooming activities ceased, tests began. The tested area was the midplantar left paw. Withdrawal responses to mechanical stimuli were determined by an electronic von Frey Anesthesiometer system (IITC Life Science, CA, USA). The electronic von Frey polypropylene tip was applied perpendicularly to the midplantar surface of the selected hind paw, and the intensity of the stimulus was automatically recorded when the paw was flexed reflexively followed by a clear flinch response after paw withdrawal. All rats were tested 5 times, with an intertest period of 15 min [26].

### 2.5. Tissue Preparation

After intrathecal injection of drugs for 24 hours, the rats were deeply anesthetized and sacrificed and lumbar enlargement was harvested for dihydroethidium (DHE) staining, terminal deoxynucleotidyl transferase-mediated deoxyuridine in situ nick end labeling (TUNEL) staining, and Western blotting. The lumbar enlargement with the anterior and posterior roots was removed en bloc from the animals. In brief, the spinal cord at L4-5 was divided into two parts, one for a frozen section the other for Western blot. The frozen section was about 15 *μ*m thick.

### 2.6. Apoptosis Assay by TUNEL Staining

Cell apoptosis was analyzed using a TUNEL detection kit (Roche, Germany) following the manufacturer's instructions. Cells were seeded on a glass slide at a concentration of 5 × 10^4^ cells/plate in a 12-well plate. SH-SY5Y cells were treated with 1.5 mM bupivacaine for 3 h and incubated with DMEM/F12 medium for 24 h after exposure. Cells were fixed by 4% paraformaldehyde for 1 h. Then, 0.1% Triton X-100 was used for 5 minutes on ice to change the permeability of cells. After rinsing by PBS, cells were covered with TdT reaction mixture for 2 h in the dark. After the final wash with PBS, fluoroshield mounting medium with DAPI (Abcam, UK) was used for mounting and nucleus staining. The protocol of TUNEL staining for a frozen section was similar as for the cells. The cells or the frozen section was examined and photographed with a fluorescence microscope. The average number of fluorescence dots of three images from each treatment group was calculated.

### 2.7. Measurement of Cytoplasmic ROS by Fluorescence Microscopy and Microplate Reader

Cells were seeded onto a piece of slide in 12-well plates at a concentration of 5 × 10^4^ cells/well in DH10. Cytoplasmic peroxide was estimated using the DCFH-DA (Sigma-Aldrich, St. Louis, MO), and cytoplasmic superoxide was estimated using the DHE (Beyotime, China) [27]. SH-SY5Y cells were treated with 1.5 mM bupivacaine for 3 hours, then incubated with 10 *μ*M DCFH-DA for 30 min or 3 *μ*M DHE for 20 min at room temperature. Stained cells were washed twice in PBS and fixed in 4% paraformaldehyde for 15 min. After the final wash by PBS, cells were treated with DAPI-Fluoromount. The cells or the frozen section was examined and photographed with a fluorescence microscope (BX51, Olympus, Japan). In microplate assay, cells were seeded in 96-well plates and stained by 10 *μ*M DCFH-DA for 30 min or 3 *μ*M DHE for 20 min before drug treatments. After the final wash by PBS, cells were examined by a microplate reader (M5, MDS, USA). The excitation wavelength of DCFH-DA is 488 nm and the emission wavelength is 525 nm. The excitation wavelength of DHE is 300 nm and the emission wavelength is 610 nm. Average fluorescence intensity of each group was calculated.

### 2.8. Immunofluorescence for Cells

The cells cultured on coverslips after bupivacaine treatment for 24 h were fixed with 4% paraformaldehyde for 15 min and washed by PBS for 3 times. Cells were incubated with 0.1% Triton X-100 to permeabilize cells. Cells were blocked with 5% BSA dissolved in PBS for 30 min, then incubated with anti-p47phox rabbit antibody (1 : 300, Thermo Fisher, USA) and anti-pan-cadherin mouse antibody (1 : 100, Abcam, UK) at 4°C overnight. On the second day, cells were washed and incubated with secondary antibody, Alexa Fluor® 488 conjugate anti-mouse antibody (Bioworld, China) and Alexa Fluor 594 conjugate anti-rabbit antibody (Bioworld, China) at a dilution of 1 : 200, for 1 h at room temperature. After washing, the cells were treated with DAPI-Fluoromount and examined using a confocal microscope and fluorescence-activated cell sorting (FACS; Nikon, TI-FL, Japan).

### 2.9. Cell Membrane Separation and Sample Protein Analysis

After treatments, cells were lysed in RIPA buffer (Sigma-Aldrich, St. Louis, MO) with 1 mM PMSF (CST, Danvers, Massachusetts, USA) and a protease/phosphatase inhibitor cocktail (CST, Danvers, Massachusetts, USA). After vortex oscillation for 5 times every 5 min, samples were centrifuged at 14000 ×g for 15 min; the supernatant (i.e., the total protein extract) was collected and the protein content was measured by Bradford test (Beyotime, China). To obtain cytosolic and membrane fractions, cells were lysed in lysis buffer provided by Keygen (Nanjing, China) with DTT and a protease inhibitor. Samples were vortex-oscillated for 5 times every 5 minutes on ice, then centrifuged at 13300 rpm for 30 min. The supernatant (cytosolic protein extract) was collected, and a pellet was dissolved in extraction buffer provided by Keygen (Nanjing, China), placed on ice for 10 min, and centrifuged at 5000 rpm for 5 min. The mixture was in a 37°C water bath for 10 minutes. Then, the mixture was centrifuged again at 13300 rpm; the membrane extraction was in the lower liquid. They were stored at −80°C and, when applicable, denaturalized in loading buffer (5 min, 95°C) just before electrophoresis.

Samples from lumbar enlargement were prepared just the same as for cells. The difference was that tissues were made into a tissue homogenate before centrifuged.

### 2.10. Western Blot Assay

Samples, including whole lysates, and cytosolic and membrane fractions from cells and tissues were subjected to Western blot analysis. A 30 *μ*g protein sample was separated by sodium dodecyl sulfate-polyacrylamide gel electrophoresis; electrotransferred to PVDF membranes; blocked with 5% BSA in TBST; and then immunoblotted with rabbit anti-p47phox (1 : 1000, Santa Cruz, USA), rabbit anti-cleaved caspase-3 (1 : 1000, CST, Danvers, Massachusetts, USA), rabbit anti-*γ*-H2Ax (1 : 1000, CST, Danvers, Massachusetts, USA), mouse anti-pan-cadherin (1 : 1000, Abcam, UK), or rabbit anti-*β*-tubulin antibody (1 : 4000, Bioworld, China) diluted in blocking solution containing 5% BSA and 0.1% Tween-20 in Tris-HCl-buffered saline overnight at 4°C. After being rinsed, membranes were incubated with HRP-conjugated anti-rabbit immunoglobulin or anti-mouse immunoglobulin at 1 : 5000 for 1 hour. Specific proteins were detected by enhanced chemiluminescence. The membranes were then exposed to X-ray film. The densities of bands were measured using a densitometer and analyzed with ImageJ. All the target protein expressions were normalized to their corresponding *β*-tubulin or pan-cadherin products.

### 2.11. Statistics

Data were analyzed by Graphpad prism ver. 5.0. All values are expressed as means ± SD. Multiple comparisons between groups in fluorescence assay, Western blot, and apoptosis assay were analyzed by one-way ANOVA. Tukey was performed as post hoc analysis for multiple comparisons between groups. Multiple comparisons between groups in paw withdrawal threshold assay were analyzed by two-way ANOVA, Bonferroni was performed as post hoc analysis for multiple comparisons between groups. A *P* < 0.05 was considered significant.

## 3. Results

### 3.1. Bupivacaine-Induced Cell Injury In Vitro and In Vivo

**Figure 1:**
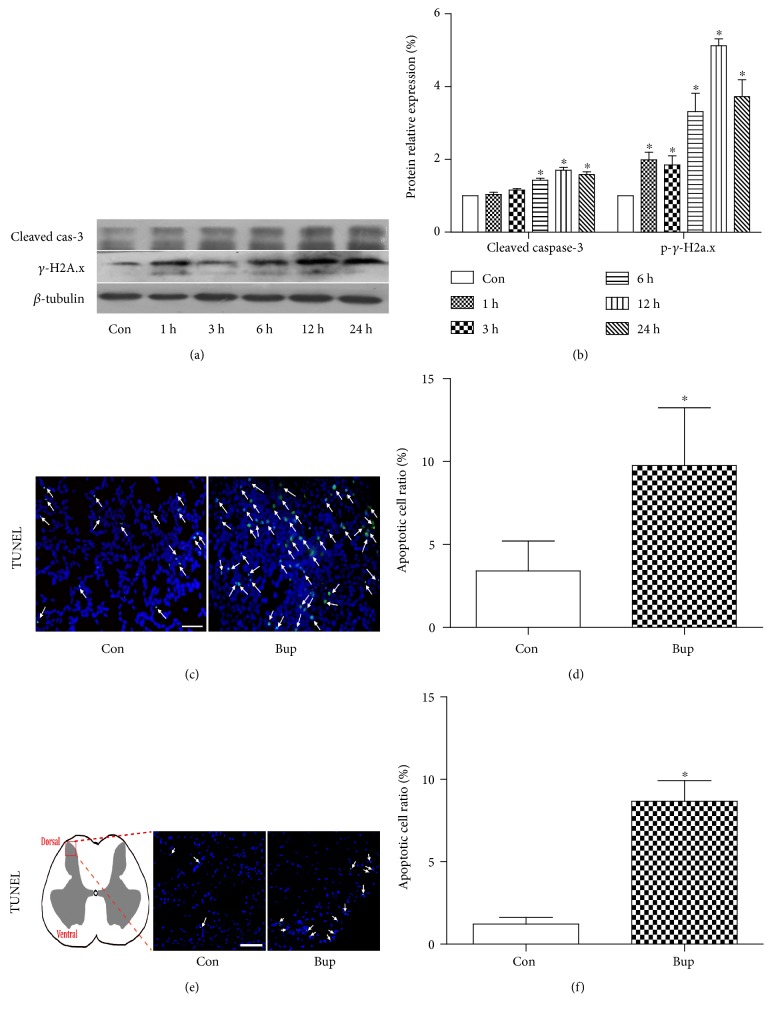
Bupivacaine-induced cell injury in vitro and in vivo. (a, b) The protein expression level of cleaved caspase-3 and phospho-*γ*-H2A.x after bupivacaine incubation at different time points. (c, d) TUNEL staining indicated the ratio of apoptotic cells after bupivacaine (1.5 mM) incubation for 24 h in SH-SY5Y cells. Cells tagged by white arrows were TUNEL positive. (e, f) Schematic diagram indicates the area of tested sections in the spinal dorsal horn. Cell apoptosis in the spinal dorsal horn was assessed by TUNEL staining. Cells tagged by white arrows were TUNEL staining positive. Data represent mean ± SD of at least 3 independent experiments or for 6 rats each group. Scale bar: 50 *μ*m.^∗^*P* < 0.05 versus control (Con) group.

As shown in Figures 1(a) and 1(b), protein expression of cleaved caspase-3 was significantly increased at 6 h, 12 h, and 24 h (*P* < 0.05 versus Con) (Figures 1(a) and 1(b)). The protein expression of *γ*-H2A.x, a marker of DNA damage, was significantly increasing starting at 1 h (*P* < 0.05 versus Con group) and peaked at 12 h (Figures 1(a) and 1(b)). Bupivacaine significantly increased cell apoptosis, as evidenced by the increase in TUNEL-positive cells in bupivacaine-treated cells (9.76 ± 3.49% versus 3.41 ± 1.79% in the control group, *P* < 0.05) (Figures 1(c) and 1(d)). In in vivo rats, the ratio of TUNEL-positive cells in the spinal cord horn in lumbar enlargement was significantly higher in the bupivacaine group than that in the control (Con) group (*P* < 0.05) (Figures 1(e) and 1(f)). Our data indicated that bupivacaine can induce cell injury both in in vitro SH-SY5Y cells and in in vivo rats.

### 3.2. NOX Inhibition Protected Cells and Rats from Bupivacaine-Induced Toxicity via Reducing Excessive Production of ROS

**Figure 2:**
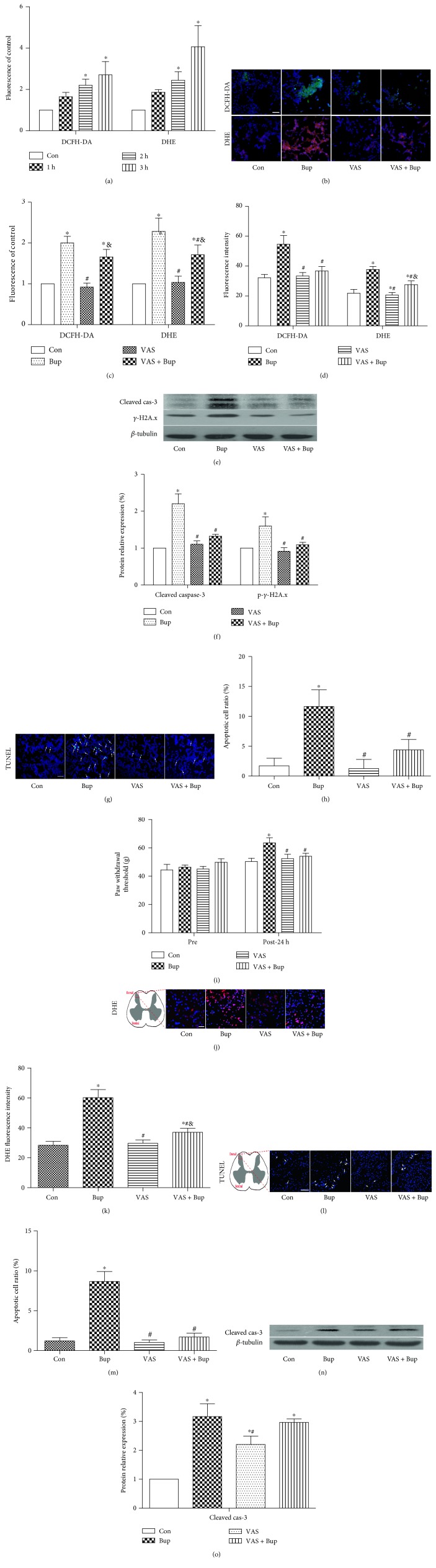
NOX inhibition protected cells and rats from bupivacaine-induced toxicity via reducing excessive production of ROS. SH-SY5Y cells were pretreated with VAS2870 (10 *μ*M) 30 min prior to bupivacaine treatment. (a) SH-SY5Y cells were treated with bupivacaine (1.5 mM) for 1 h, 2 h, and 3 h; the production of cytoplasmic peroxide (DCFH-DA) and O_2_^.−^ (DHE) was measured by a microplate reader. (b, c) SH-SY5Y cells were treated with bupivacaine for 3 h. The level of cytoplasmic peroxide (DCFH-DA, green) and O_2_^.−^ (DHE, red) was measured by microscopy. (d) The production of cytoplasmic peroxide (DCFH-DA) and O_2_^.−^ (DHE) was measured by a microplate reader. (e, f) The protein level of cleaved caspase-3 and phospho-*γ*-H2A.x after bupivacaine treatment. (g, h) The ratio of apoptotic cells was detected by TUNEL staining. Cells tagged by white arrows were TUNEL positive. (i) The basic lines of paw mechanical thresholds were tested before treatment. Paw mechanical threshold was tested by the electronic von Frey system after different treatments. (j, k) Schematic diagram indicates the area of tested sections in the spinal dorsal horn. The level of cytoplasmic O_2_^.−^ (DHE, red) was measured by microscopy. (l, m) The ratio of apoptotic cells was detected by TUNEL staining. Cells tagged by white arrows were TUNEL positive. (n, o) The protein level of cleaved caspase-3 was measured by Western blot. Data represent mean ± SD of at least 3 independent experiments or for 6 rats each group. Scale bar: 50 *μ*m. ^∗^*P* < 0.05 versus control group. ^#^*P* < 0.05 versus Bup group. ^&^*P* < 0.05 versus VAS group.

SH-SY5Y cells were exposed to 1.5 mM bupivacaine for 1 h, 2 h, and 3 h, before measurements of cytoplasmic O_2_^.−^ and cytoplasmic peroxide using DHE and DCFH-DA staining. As shown in Figure 2(a), exposure to 1.5 mM bupivacaine produced excessive cytoplasmic O_2_^.−^ and peroxide in a time-dependent manner as compared to the control group without bupivacaine exposure. NOX inhibition with VAS2870 pretreatment significantly reduced the production of cytoplasmic O_2_^.−^ (*P* < 0.05, as shown in Figures 2(b) and 2(c)) and reduced the production of cytoplasmic peroxide (not significantly) that was associated with the reductions in protein expression of cleaved caspase-3 and *γ*-H2A.x and the ratio of TUNEL-positive cells (*P* < 0.05 versus Bup, Figures 2(e), 2(f), 2(g), and 2(h)). In in vivo rats, paw withdrawal threshold was significantly increased in the bupivacaine-treated group accompanied by enhanced cytoplasmic O_2_^.−^ production and cell apoptosis (increase in TUNEL-positive cells and elevation of cleaved caspase-3 protein expression) (*P* < 0.05 versus Con, Figures 2(i), 2(j), 2(k), 2(l), 2(m), 2(n), and 2(o)). All these changes were reversed by NOX inhibition with VAS2870 (*P* < 0.05, as shown in Figures 2(i), 2(j), 2(k), 2(l), 2(m), 2(n), and 2(o). Our results showed that bupivacaine-induced cell injury was highly associated with overproduction of ROS and reducing excessive ROS formation by NOX inhibition with VAS2870 had protective effect against bupivacaine-induced toxicity both in vitro and in vivo.

### 3.3. Membrane Translocation of p47phox Was Increased after Bupivacaine Exposure Which Was Inhibited by NOX Inhibition

**Figure 3:**
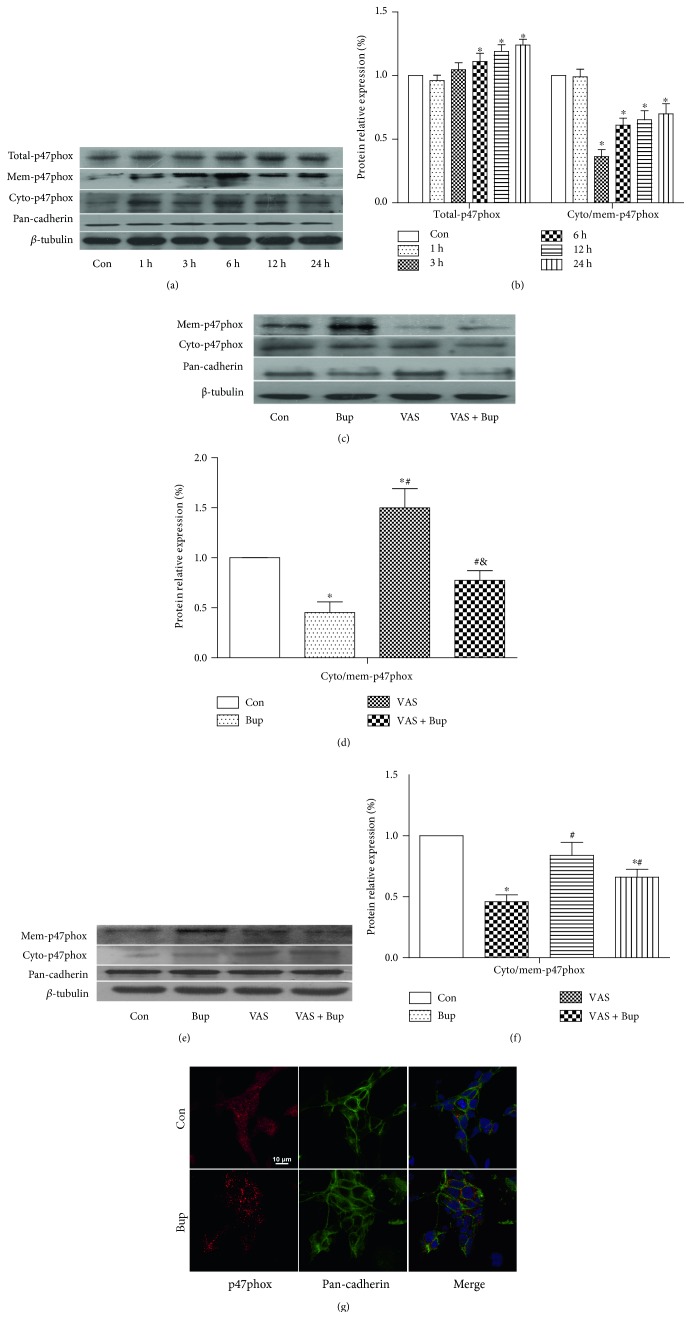
Membrane translocation of p47phox was increased after bupivacaine exposure, which was inhibited by NOX inhibition. (a, b) The protein level of total p47phox after bupivacaine incubation at different time points was measured by Western blot. The level of p47phox membrane translocation was detected by the ratio of p47phox expressed in the cytosol and membrane. (c, d) SH-SY5Y cells were treated with 1.5 mM bupivacaine for 3 h and incubated with DH10 medium until 24 h. SH-SY5Y cells were pretreated with VAS2870 (10 *μ*M) 30 min prior to bupivacaine treatment. The level of p47phox translocation in SH-SY5Y cells with different treatments. (e, f) The level of p47phox translocation in animal models. (g) The level of p47phox membrane translocation was detected by immunofluorescent staining. Pan-cadherin is a widely used membrane marker and was stained green, targeted protein p47phox was stained red, and nucleus was stained with DAPI (blue). Data represent mean ± SD of at least 3 independent experiments or for 6 rats each group. Scale bar: 10 *μ*m.^∗^*P* < 0.05 versus Con group. ^#^*P* < 0.05 versus Bup group. ^&^*P* < 0.05 versus VAS group.

Cytoplasmic ROS is generated by the activated NOX, and p47phox is considered to be the initial and essential factor of NOX2 activation. As shown in Figure 3, in SH-SY5Y cells, the level of p47phox is examined by the ratio of p47phox expressed in the cytosol and membrane [22, 23, 28, 29]. As shown in Figures 3(a) and 3(b), p47phox expressed in the whole cell lysate was upregulated in a time-dependent manner which peaked at 24 hours. In groups of 3 h, 6 h, 12 h, and 24 h, the cytosol/membrane ratio of p47phox was significantly decreased with the largest decline in the 3 h group (*P* < 0.05, Figures 3(a) and 3(b)). These were confirmed in the double immunofluorescent staining of p47phox (red) and pan-cadherin (green, a recognized cell membrane marker) which showed that bupivacaine-treated cells had more red merged with green (Figure 3(g)). However, this bupivacaine-induced activation of p47phox was inhibited by NOX inhibition with VAS2870 (*P* < 0.05, Figures 3(c) and 3(d)). In in vivo rats, membrane translocation of p47phox was significantly increased in bupivacaine-treated rats, which was reduced by NOX inhibition with VAS2870 (*P* < 0.05, Figures 3(e) and 3(f)). Our results suggested that membrane translocation of p47phox was highly associated with bupivacaine-induced cell injury and that pretreatment with NOX inhibition with VAS2870 reduced the membrane translocation of p47phox and attenuated bupivacaine-induced cell injury.

### 3.4. p47phox Gene Knockdown Reduced Bupivacaine-Induced ROS Overexpression and Cell Injury

**Figure 4:**
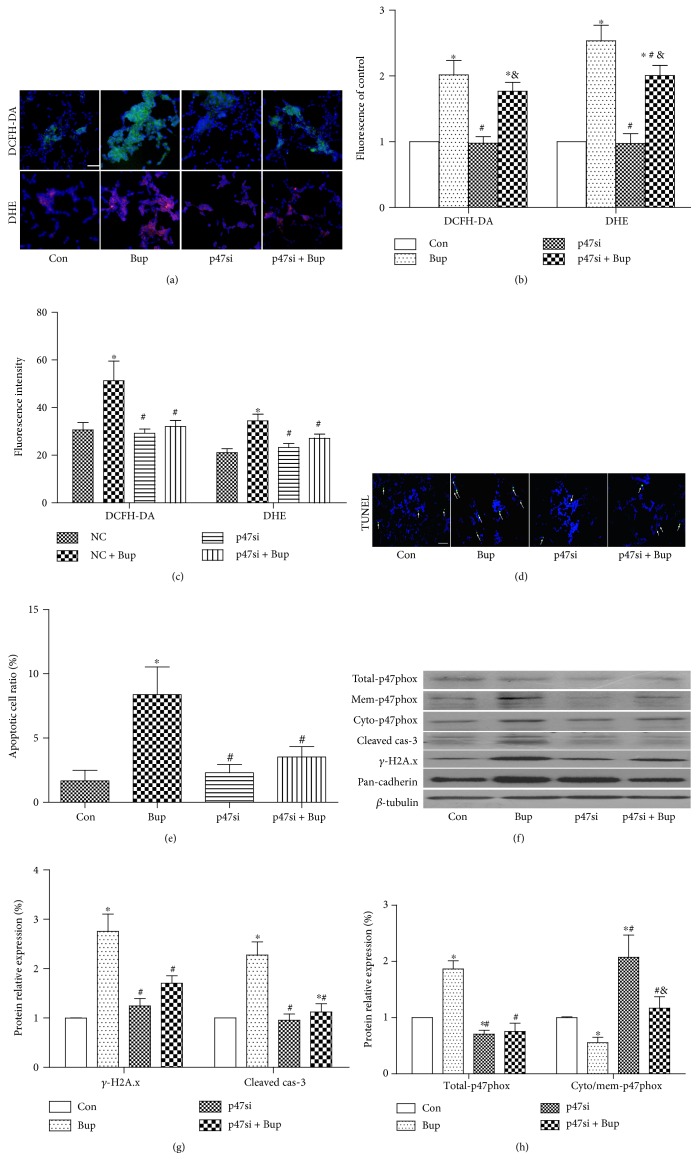
p47phox gene knockdown reduced bupivacaine-induced ROS overexpression and cell injury. (a, b) The level of cytoplasmic peroxide (DCFH-DA, green) and O_2_^.−^ (DHE, red) was measured by microscopy. (c) The production of cytoplasmic peroxide (DCFH-DA) and O_2_^.−^ (DHE) was measured by a microplate reader. (d, e) The ratio of apoptotic cells was detected by TUNEL staining. Cells tagged by white arrows were TUNEL positive. (f, g) The protein level of cleaved caspase-3 and phospho-*γ*-H2A.x was measured by Western blot. (f, h) The translocation of p47phox was measured by Western blot. Data represent mean ± SD of at least 3 independent experiments. Scale bar: 50 *μ*m. ^∗^*P* < 0.05 versus control (NC) group. ^#^*P* < 0.05 versus Bup group. ^&^*P* < 0.05 versus p47si group.

In order to confirm the role of p47phox in bupivacaine-induced cell injury, p47phox was knocked down by siRNA in SH-SY5Y cells in the presence or absence of bupivacaine. The results showed that knockdown of p47phox decreased cytoplasmic O_2_^.−^, reduced cytoplasmic peroxide (not significantly), downregulated cell apoptosis (reduced TUNEL-positive cells and decreased cleaved caspase-3 protein expression), and decreased *γ*-H2A.x protein expression in bupivacaine-treated cells (*P* < 0.05, Figures 4(a), 4(b), 4(c), 4(d), 4(e), 4(f), and 4(g)). p47phox gene knockdown resulted in 30% reduction in p47phox in the cells and decreased the membrane translocation of p47phox in bupivacaine-treated cells (*P* < 0.05, Figures 4(f), 4(g), and 4(h)). DCFH-DA is a widely used fluorescent probe for ROS. Price and Kessel [30] reported that the substances detected by DCFH-DA are varied and not highly selective. The results in the present study showed that transfection with p47phox-siRNA did not lower the production of peroxide detected by DCFH-DA. It may be associated with idea that the produced substance detected by DCFH-DA was not the predominant substance in a bupivacaine-treated cell model. Our results indicated that p47phox gene knockdown inhibited the membrane translocation of p47phox thereby reducing bupivacaine-induced ROS overproduction and cell injury.

## 4. Discussion

There are three main findings in the present study. First, bupivacaine induced neuron cell injury both in vitro and in vivo through overproduction of ROS. Second, bupivacaine could induce membrane translocation of p47phox to activate NOX to produce excessive ROS, resulting in cell injury. Third, inhibition of NOX, in particular p47phox, attenuated bupivacaine-induced excessive ROS production and cell injury. Collectively, our results indicated that bupivacaine induced neuron cell injury by inducing excessive ROS production, and it does so through enhancing membrane translocation of p47phox and the subsequent activation of NOX.

Bupivacaine, an amide-type local anesthetic, is one of the most widely used local anesthetics in clinics. We previously found that bupivacaine can induce excessive ROS production and apoptosis in SH-SY5Y cells [5, 11, 12]. However, the mechanism whereby bupivacaine induced ROS and cell apoptosis in SH-SY5Y cells remains unclear. In the current study, we demonstrated that bupivacaine treatment increased ROS production and apoptosis in SH-SY5Y cells that were associated with increased p47phox activation (increased membrane translocation), while pharmacological inhibition or gene knockdown of p47phox attenuated bupivacaine-induced ROS and cell injury. These indicated that bupivacaine induced ROS and cell injury through increasing the level of membrane translocation of p47phox. It has been well accepted that apoptosis can be triggered by ROS, RNS (reactive nitrogen species), DNA-damaged reagents, and so on [14]; in addition, ROS has been shown to enhance RNS and DNA damage and activate the upstream of caspase family to cleave the downstream caspase effector, such as caspase-3 [14]. Together, these suggest that in the current study, bupivacaine by enhancing p47phox, which subsequently induced ROS overexpression and thereby activated apoptosis, resulted in cell injury.

Reductive stress, defined as an excessive reduction in ROS by antioxidant agents in a cell that leads to shortage of ROS, has been shown to be harmful to cells, which has been suggested as the reason why no positive results of antioxidant therapies were gained from clinical studies [24]. This provides a clue that targeting the source of ROS may be a new strategy for combating oxidative stress and its related diseases. NOX-derived ROS is the initiate and physiologic source of ROS. Only when NOX2 is activated, cytoplasmic O_2_^.−^ is produced [13]. In general, activation of NOX2 requires translocation of cytoplasmic factors to membrane subunit. The p47phox consists of a PX domain which interacts with membrane phosphatidic acid (PA) and membrane lipids [phosphatidylinositol-(3,4)-bisphosphate [PI(3,4)P2]. p47phox is tethered to the membrane subunits by directly interacting with the proline-rich region (PRR) of p22phox through Src homology 3 (SH3) domains. There is an autoinhibitory region (AIR) and a C-terminal PRR domain for interaction with other NADPH oxidase subunits [20, 21]. In the phagosome system [20], when NOX2 is inactivated, the p47phox, p67phox, and p40phox are in the cytoplasm. p47phox is folded and inactive due to AIR. In activation of NOX2, p47phox is initially phosphorylated by serine residuals via the PKC-dependent pathway, resulting in AIR destabilization which exposes the PX domain and SH3 domain to membrane lipids PI(3,4)P2, PA, and the PRR of p22phox [21, 28]. The movement of p47phox brings other cytosolic subunit, to form the active NOX2. The activated NOX2 generates O_2_^.−^ and peroxide [13]. In a cell-free system, single mutation of serine residual 379 almost abrogated p47phox translocation to the membrane and interaction with p22phox [28]. Moreover, in other cells, phosphorylation and translocation of p47phox are the most important elements in NOX2 activation-induced ROS burst. The p47phox protein variant expression Ncf1^m1J^ mice was reported as completely defective in activating the NOX2 complex to produce ROS, and at the same time, p47phox failed to translocate to the membrane [29]. In ZDF rats, inhibiting p47phox membrane translocation led to inhibition of NOX activity [22]. In our study, membrane translocation of p47phox was significantly increased after 3 hours of exposure of bupivacaine, which was consistent with the elevation of the cellular ROS, while reducing p47phox membrane translocation by pharmacological inhibition or gene knockdown of p47phox reduced bupivacaine-induced ROS and cell injury, indicating that p47phox plays an important role in bupivacaine-induced ROS and cell injury.

In the current study, we demonstrated that p47phox plays a critical role in bupivacaine-induced ROS and cell injury in our in vivo rat models and in vitro cell models, suggesting that inhibition of p47phox-mediated NOX activation may serve as a therapeutic strategy to protect cells against bupivacaine-induced cell injury. Indeed, in our in vivo rat model, we provided evidences that inhibition of NOX with VAS2870 attenuated bupivacaine-induced neuron cell injury. The NOX inhibitor VAS2870, as we used in our study, is a triazolopyrimidine that is developed by the company Vasopharm GmbH in a screening approach for NOX2 inhibitors. It is established as an effective inhibitor for NOX1, NOX2, and NOX4 [15]. VAS2870 has not been shown to be intrinsic antioxidant, as evidenced by its inability to affect superoxide anion levels in the xanthine/xanthine oxidase assay. Moreover, it was found to be noncytotoxic [31]. Compared to the traditional inhibitor apocynin, VAS2870 is more specific to NOX-derived ROS [15]. As compared to a small peptide gp91dstat, VAS2870 had a better bioavailability [15, 32]. VAS2870 only inhibited NOX activity when added before the induction of NOX2 active complex assembly but not when added after complex assembly [24]. VAS2870 inhibited the production of cellular O_2_^.−^ but showed no effect on semirecombinant NOX2 assay and translocation of p47phox [33]. But in vascular smooth muscle cells (VSMC), VAS2870 showed a significant effect on inhibiting NOX activity [31]. VAS2870 was reported to be protective against ischemic stroke as a NOX inhibitor [25, 34]. Most of the studies confined to the cell-free system and human neutrophil and endothelial systems. The ability of NOX activity inhibition is of consensus. But the mechanism is underdetermined. In our study, pretreatment with VAS2870 did reduce the production of cytoplasmic ROS and cell injury in SH-SY5Y cells. In the rat model, VAS2870 had a significant effect on declining the increase in withdrawal threshold, the level of cytoplasmic O_2_^.−^, and TUNEL staining-positive cells in the spinal dorsal horn. In both cell and animal models, p47phox membrane translocation was inhibited. Our results suggest that VAS2870 protected SH-SY5Y cells and rats from bupivacaine-induced ROS overproduction via inhibiting p47phox translocation.

In conclusion, bupivacaine induced membrane translocation of p47phox, which activated NOX, resulting in burst production of ROS and thereby inducing cell injury. Inhibition of NOX or gene knockdown of p47phox decreased bupivacaine-induced ROS overproduction and attenuated bupivacaine-induced cell injury.

## Abbreviations

NOX: NADPH oxidase
ROS: Reactive oxygen species
*γ*-H2A.x: Phospho-histone H2A.x.
